# Current Landscape of Generative AI Use as a Search Engine Among Resident Physicians: Cross-Sectional Study

**DOI:** 10.2196/89750

**Published:** 2026-07-17

**Authors:** Toshiki Miwa, Koh Okamoto, Yuji Nishizaki, Yasuharu Tokuda

**Affiliations:** 1 Department of Infectious Diseases, University of Tokyo Hospital, Tokyo, Japan; 2 Department of Infectious Diseases, Graduate School of Medical and Dental Sciences, Institute of Science Tokyo, 1-5-45 Yushima, Bunkyo-ku, Tokyo, 113-8519, Japan, 81 3-5803-4138, 81 3-5803-0157; 3 Division of Medical Education, Faculty of Medicine, Juntendo University, Tokyo, Japan; 4 Muribushi Okinawa Center for Teaching Hospitals, Urasoe, Okinawa, Japan

**Keywords:** postgraduate education, generative AI, medical ethics, information literacy, Japan

## Abstract

**Background:**

Acquiring generative AI (GenAI) literacy and avoiding knowledge collapse and inadequate skill development are critical for resident physicians in the GenAI era, who are highly susceptible to its influence, including those who are current nonusers.

**Objective:**

This study examined the degree of GenAI use as a search engine in clinical practice among resident physicians and compared differences in AI literacy and bedside behaviors in the management of infectious diseases between GenAI users and nonusers.

**Methods:**

This cross-sectional study was conducted through an original questionnaire survey administered in January 2025 to participants of the General Medicine In-Training Examination (GM-ITE), a nationwide computer-based test for postgraduate year 1 and year 2 resident physicians in Japan. The survey items covered the degree of GenAI use, GenAI literacy, and perceived bedside behaviors. In addition to the GM-ITE score, we collected data on demographics and survey responses. Statistical analysis was performed to compare attitudes toward GenAI use between GenAI users and nonusers. Additionally, the difference in their bedside behaviors in infectious disease management was assessed, adjusted for demographics, the learning environment, and GM-ITE score.

**Results:**

Of 9179 GM-ITE examinees, 2989 (32.6%) resident physicians from 546 hospitals completed the survey. Subsequent analyses were performed in 2850 respondents who answered the question regarding GenAI use as a search engine in clinical work. A total of 1124 (39.4%) participants reported using GenAI as a search engine, while 1726 (60.6%) were nonusers. Only 13% (144/1110) of users relied on it as a primary reference for differential diagnoses. Compared with nonusers, users were more likely to understand limitations such as confabulation (817/1103, 74.1% vs 991/1696, 58.4%; *P*<.001) and to recognize competencies essential for future physicians in infectious disease management, including history taking and physical examination (965/1106, 87.3% vs 1296/1706, 76%; *P*<.001). Ethical considerations were less commonly addressed overall, with 47.3% (1311/2770) examining fairness and 49.8% (1381/2774) examining transparency. Awareness was greater among users than nonusers (615/1092, 56.3% vs 696/1678, 41.5%; *P*<.001 for fairness and 645/1095, 58.9% vs 736/1679, 43.8%; *P*<.001 for transparency). GenAI use was independently associated with perceived favorable behaviors, such as thorough examination (adjusted odds ratio [aOR] 1.42, 95% CI 1.18‐1.70) and appropriate antimicrobial use (aOR 1.57, 95% CI 1.32‐1.87).

**Conclusions:**

Although GenAI use was not associated with self-reported behaviors indicative of knowledge collapse at this stage, GenAI literacy among resident physicians in Japan remains limited, particularly among nonusers. Given its rapid expansion, education on GenAI in clinical practice may be necessary for both users and nonusers, emphasizing critical thinking and ethical considerations, including transparency, in patient care.

## Introduction

In recent years, generative AI (GenAI) has gained significant attention and has been increasingly integrated into clinical care. GenAI is expected to serve as an “interactive encyclopedia,” supporting clinical decisions by providing disease information, listing differential diagnoses, and suggesting treatment options at a level comparable to those of physicians [1-4]. However, its inherent limitations, stemming from its stochastic nature and training data, include confabulations and biases [5]. These limitations are compounded by human cognitive bias toward GenAI outputs [2,6].

Concerns around knowledge collapse and inadequate skill development (eg, deskilling, never-skilling, and mis-skilling) among physicians who overrely on GenAI are also emerging [2,7,8]. They are theoretically caused by cognitive off-loading and have already been documented in endoscopists [9]. We hypothesized that a similar phenomenon may be observed in other competencies, including those that remain indispensable even when using GenAI. For example, clinical reasoning grounded in pathophysiological knowledge is essential for identifying confabulations [8,10]. Similarly, considering local disease epidemiology is crucial to counter biases embedded in large databases [6]. Moreover, thorough history taking and physical examination skills are unlikely to be replaced by GenAI [11,12]. The importance of these competencies is particularly true for infectious diseases (IDs), where bedside evaluation and awareness of local pathogen epidemiology and antimicrobial resistance are central to diagnosis and treatment but are insufficiently represented in current GenAI models [6].

Resident physicians’ recognition of these drawbacks is of paramount importance. First, as digital natives, they may be less critical of advanced technologies [13]. Second, with limited clinical experience, they may struggle to evaluate the accuracy of GenAI outputs [13]. Third, their clinical performance has enduring implications for patient care. These concerns extend to GenAI nonusers, given that the adoption of GenAI in medicine is unlikely to cease [2]. However, the preparedness of nonusers for its future integration remains unclear.

This study aimed to explore the current landscape of (1) the resident physicians’ use of GenAI in clinical practice, (2) awareness of GenAI’s limitations among users and nonusers, and (3) differences in bedside behaviors regarding the management of IDs between the two groups to assess knowledge collapse and inadequate skill development among GenAI users.

## Methods

### Study Setting, Study Participants, and Data Source

We conducted a cross-sectional study in January 2025 among examinees of the General Medicine In-Training Examination (GM-ITE) in Japan, where the academic year runs from April to March. The GM-ITE is a nationwide, annual computer-based test consisting of 80 multiple-choice clinical questions for postgraduate year (PGY) 1 and PGY 2 resident physicians. It has been administered by the Japan Institute for Advancement of Medical Education Program (JAMEP), a nonprofit organization that evaluates medical education in Japan, since 2011. In 2024, 9580 resident physicians—approximately half of all PGY 1 and PGY 2 residents in Japan—took the examination [14]. GM-ITE data have been widely used in medical education research [15-19].

We developed a 29-item multiple-choice electronic survey addressing GenAI use in daily life outside clinical work, GenAI use as a search engine, and bedside behaviors in ID management (Multimedia Appendix 1), which was disseminated concurrently with the examination. The variable regarding GenAI use in daily life was collected to provide contextual information on study participants. Participation was voluntary, and respondents received an online reference list of common pathogens and antimicrobials, which did not contain GenAI-related content, as an incentive. All GM-ITE examinees who responded to our survey and consented to the use of their responses for research purposes were included in our study. For the comparison between resident physicians with and without GenAI use as a search engine, we analyzed responses only from individuals who responded to the relevant item.

In Japan, 2-year residency programs start in April and end in March. They are mandatory for all medical school graduates seeking clinical positions, regardless of their desired specialty. An ID department rotation is elective, whereas an antimicrobial stewardship program is not included [15].

### Questionnaire Development

GenAI use as a search engine in daily life and in clinical work was assessed using separate survey items. We designed the survey to evaluate respondents’ GenAI literacy using Bloom’s taxonomy of learning domains, a framework commonly applied in medical education [20]. The framework comprises the psychomotor (eg, practical skills), cognitive (eg, knowledge), and affective (eg, attitudes or medical ethics) domains. In the psychomotor domain, we investigated whether a respondent used GenAI to gain information about the disease of interest or to help enumerate differential diagnoses. The cognitive domain examined perceived GenAI literacy, including confabulation [6,21], biased output [6], automation bias [2,6], physicians’ knowledge collapse and deskilling [2,8], and uninformed use of GenAI in patient care [22], as a surrogate for true GenAI literacy. It also addressed competencies considered essential regardless of GenAI, such as history taking, physical examination [11,12], pathophysiological knowledge, and clinical reasoning [8,10]. The affective domain adopted the FAVES (fairness, appropriateness, validity, effectiveness, and safety) framework and transparency, an expansion of the Belmont principles (ie, beneficence, respect for people, and justice), to assess ethical considerations of GenAI [22].

Furthermore, we also included items on bedside behaviors crucial to ID management and likely to remain essential, including history taking, physical examination, clinical reasoning, recognition of local epidemiology (ie, epidemics and antibiogram data), and individualization of care goals [6,8,10-12]. We piloted the survey among resident physicians at the University of Tokyo Hospital to evaluate clarity and feasibility. After administration of the GM-ITE, demographic information and survey responses were collected.

### Statistical Analysis

In statistical hypothesis testing, responses on a 5-point Likert scale were dichotomized: scores 1 to 3 (strongly disagree, disagree, and neutral) were categorized as “not applicable” and scores 4 to 5 (agree and strongly agree) as “applicable” and then summarized as proportions, including the items assessing GenAI use as a search engine and bedside behaviors. Associations between GenAI use and respondents’ demographics and cognitive and affective domains were assessed using univariable chi-square tests.

Our previous study suggested the potential independent effect of an ID department rotation and the presence of antimicrobial stewardship programs on the performance of resident physicians in managing IDs [15]. Therefore, this study used multivariable logistic regression to examine associations between GenAI use (independent variable) and bedside behaviors (dependent variables), adjusting for these two factors, alongside sex, PGY, institution type (ie, university or community hospital), and overall GM-ITE score.

We performed a sensitivity analysis on the cutoff of item responses, wherein 5-point Likert scale items were dichotomized into scores 1 to 2 and scores 3 to 5. This shift also applied to the cutoff used to distinguish between GenAI users and nonusers. Statistical significance was defined as a 2-tailed *P*<.05. Throughout the statistical testing, complete-case analyses were conducted because the proportion of missing values remained below 5% [23]. Analyses were conducted using Stata (version 16; StataCorp LLC).

### Ethical Considerations

This study was approved by the Institutional Review Board of JAMEP (number 24‐20). All survey responses were examined after obtaining informed consent from the study participants. The study data were deidentified, and no identification of individual participants is possible in any portion of the manuscript or supplementary material. The study adhered to the STROBE (Strengthening the Reporting of Observational Studies in Epidemiology) reporting guidelines.

## Results

### Characteristics of Study Participants

Of 9179 GM-ITE participants, 2989 (32.6%) resident physicians from 546 hospitals completed the survey, including 1002 (33.5%) female respondents. Respondents comprised 1506 (50.4%) PGY 1 and 1483 (49.6%) PGY 2, with 2701 (90.4%) aged <30 years. Most respondents (n=2401, 80.3%) worked in community hospitals. Overall, 1948 (68.4%) reported using GenAI in daily life outside clinical work.

After excluding 139 (4.3%) resident physicians who did not respond to the item regarding GenAI use as a search engine, the remaining 2850 respondents were included in subsequent analyses. Differences between GenAI users (n=1124, 39.4%) and nonusers (n=1726, 60.6%) are summarized in Table 1. On the basis of the proportion of GenAI users among each sex, female respondents were less likely than male respondents to use GenAI as a search engine (350/963, 36.3% vs 774/1887, 41%; *P*=.02). GenAI users relied on self-study more frequently than nonusers (759/1118, 67.9% vs. 981/1706, 57.5%; *P*<.001). No statistically significant differences were observed in the proportion of PGY 1 residents (*P*=.72), individuals aged <30 years (*P*=.40), or individuals working at a university hospital (*P*=.84).

**Table 1.** Association between the use of generative AI (GenAI) as a search engine and resident physicians’ demographics and educational resources (N=2850^a^).

Variables	Overall (n=2850), n (%)	GenAI users (n=1124), n (%)	GenAI nonusers (n=1726), n (%)	*P* value
Participants demographics				
Sex				.02
Male	1887 (66.2)	774 (68.9)	1113 (64.5)	
Female	963 (33.8)	350 (31.1)	613 (35.5)	
PGY^b^				.72
PGY 1	1424 (50)	557 (49.6)	867 (50.2)	
PGY 2	1426 (50)	567 (50.4)	859 (49.8)	
Age <30 y (n=2714)^c^	2435 (89.7)	971 (90.3)	1464 (89.3)	.40
Institution type				.84
University hospital	548 (19.2)	214 (19)	334 (19.4)	
Community hospital	2302 (80.8)	910 (81)	1392 (80.6)	
Educational resources				
I have received training on the application of GenAI as a search engine (n=2837)^d^.	339 (12)	210 (18.8)	129 (7.5)	<.001
I rely on self-study instead of seeking advice from an attending physician to address questions (n=2824)^e^.	1740 (61.6)	759 (67.9)	981 (57.5)	<.001
What resources do you primarily use to address questions concerning unfamiliar diseases? (n=2827)^f^				
Printed or electronic textbooks	1150 (40.7)	419 (37.7)	731 (42.6)	—^g^
Medical apps	762 (27)	310 (27.9)	452 (26.4)	—
Internet search without using GenAI	636 (22.5)	216 (19.4)	420 (24.5)	—
GenAI-generated articles or summaries	168 (5.9)	117 (10.5)	51 (3)	—
Review articles	74 (2.6)	32 (2.9)	42 (2.4)	—
Original articles	37 (1.3)	18 (1.6)	19 (1.1)	—
What resources do you primarily use to address questions concerning familiar diseases? (n=2819)^h^				
Printed or electronic textbooks	1567 (55.6)	592 (53.3)	975 (57.1)	—
Medical apps	652 (23.1)	255 (23)	397 (23.2)	—
Internet search without using GenAI	399 (14.2)	134 (12.1)	265 (15.5)	—
GenAI-generated articles or summaries	134 (4.8)	97 (8.7)	37 (2.2)	—
Review articles	49 (1.7)	23 (2.1)	26 (1.5)	—
Original articles	18 (0.6)	10 (0.9)	8 (0.5)	—
What resources do you primarily use to address differential diagnoses? (n=2817)^i^				
Printed or electronic textbooks	1611 (57.2)	600 (54.1)	1011 (59.2)	—
Medical apps	579 (20.6)	210 (18.9)	369 (21.6)	—
Internet search without using GenAI	393 (14)	129 (11.6)	264 (15.5)	—
GenAI-generated articles or summaries	188 (6.7)	144 (13.0)	44 (2.6)	—
Review articles	32 (1.1)	20 (1.8)	12 (0.7)	—
Original articles	14 (0.5)	7 (0.6)	7 (0.4)	—

a Responses from some study participants are missing.

b PGY: postgraduate year.

c Overall: n= 2714; GenAI users: n=1075; GenAI nonusers: n=1639.

d Overall: n= 2837; GenAI users: n=1120; GenAI nonusers: n=1717.

e Overall: n= 2824; GenAI users: n=1118; GenAI nonusers: n=1706.

f Overall: n= 2827; GenAI users: n=1112; GenAI nonusers: n=1715.

g Statistical testing is not applicable.

h Overall: n= 2819; GenAI users: n=1111; GenAI nonusers: n=1708.

i Overall: n= 2817; GenAI users: n=1110; GenAI nonusers: n=1707.

### Psychomotor Domain of GenAI Use Among Resident Physicians

Among all respondents (n=2850), 344 (12.1%) reported receiving training in GenAI use as a search engine. GenAI was infrequently used as the primary educational resource: 13% (144/1110) of respondents used it for differential diagnoses, 10.5% (117/1112) for unfamiliar diseases, and 8.7% (97/1111) for familiar diseases. Even among GenAI users, printed or electronic textbooks remained the predominant educational resource: 54.1% (600/1110) for differential diagnoses, 53.3% (592/1111) for familiar diseases, and 37.7% (419/1112) for unfamiliar diseases.

### Cognitive Domain of GenAI Use Among Resident Physicians

**Figure 1.**
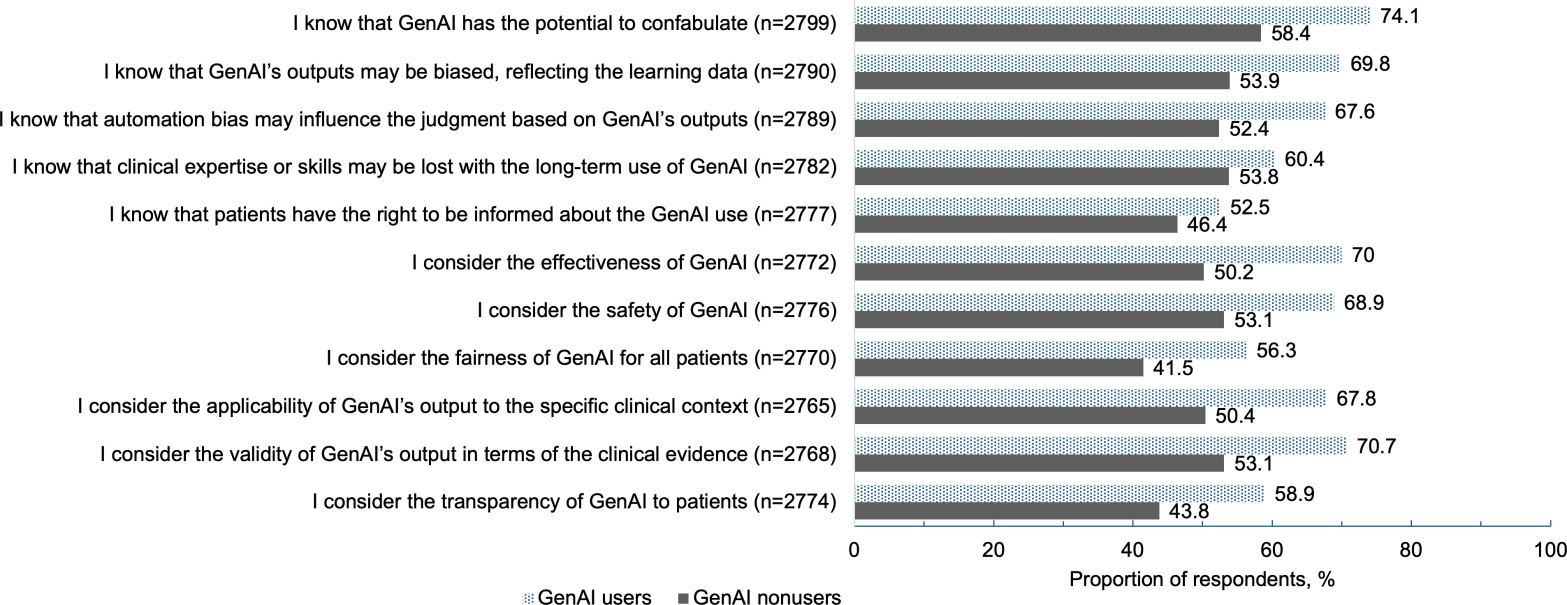
Resident physicians’ perceived generative AI (GenAI) literacy in cognitive and affective domains.

The distribution of responses for each item on 5-category scales was illustrated in Multimedia Appendix 2. GenAI’s potential for confabulation and bias was recognized by 64.6% (1808/2799) and 60.2% (1679/2790) of respondents, respectively, while only 48.8% (1356/2777) acknowledged patients’ right to be informed of GenAI use. Awareness was consistently higher among GenAI users than nonusers (Table 2 and Figure 1): confabulation (817/1103, 74.1% vs 991/1696, 58.4%; *P*<.001), biased output (769/1101, 69.8% vs 910/1689, 53.9%; *P*<.001), automation bias (743/1099, 67.6% vs 885/1690, 52.4%; *P*<.001), knowledge collapse (664/1099, 60.4% vs 905/1683, 53.8%; *P*=.001), and informing patients of GenAI use (578/1102, 52.5% vs 778/1675, 46.4%; *P*=.002).

**Table 2.** Association between the use of generative AI (GenAI) as a search engine and resident physicians’ perceived GenAI literacy in cognitive and affective domains (n=2850^a^).

Variables	Overall, n (%)	GenAI users, n (%)	GenAI nonusers, n (%)	*P* value
Cognitive domain				
I know that GenAI has the potential to confabulate (n=2799)^b^	1808 (64.6)	817 (74.1)	991 (58.4)	<.001
I know that GenAI outputs may be biased, reflecting the learning data (n=2790)^c^	1679 (60.2)	769 (69.8)	910 (53.9)	<.001
I know that automation bias may influence the judgment based on GenAI outputs (n=2789)^d^	1628 (58.4)	743 (67.6)	885 (52.4)	<.001
I know that clinical expertise or skills may be lost with the long-term use of GenAI (n=2782)^e^	1569 (56.4)	664 (60.4)	905 (53.8)	.001
I know that patients have the right to be informed about GenAI use (n=2777)^f^	1356 (48.8)	578 (52.5)	778 (46.4)	.002
I think that history taking and physical examination remain crucial in the management of IDs^g^ (n=2812)^h^	2261 (80.4)	965 (87.3)	1296 (76)	<.001
I think that understanding pathophysiology remains crucial in the management of IDs (n=2799)^i^	2244 (80.2)	958 (86.9)	1286 (75.8)	<.001
I think that clinical reasoning ability remains crucial in the management of IDs (n=2795)^j^	2252 (80.6)	951 (86.2)	1301 (76.9)	<.001
Affective domain				
I consider the effectiveness of GenAI (n=2772)^k^	1607 (58)	764 (70)	843 (50.2)	<.001
I consider the safety of GenAI (n=2776)^l^	1647 (59.3)	755 (68.9)	892 (53.1)	<.001
I consider the fairness of GenAI for all patients (n=2770)^m^	1311 (47.3)	615 (56.3)	696 (41.5)	<.001
I consider the applicability of GenAI’s output to the specific clinical context (n=2765)^n^	1584 (57.3)	740 (67.8)	844 (50.4)	<.001
I consider the validity of GenAI’s output in terms of the clinical evidence (n=2768)^o^	1663 (60.1)	772 (70.7)	891 (53.1)	<.001
I consider transparency regarding GenAI use with patients (n=2774)^p^	1381 (49.8)	645 (58.9)	736 (43.8)	<.001

a Responses from some study participants are missing.

b Overall: n= 2799; GenAI users: n=1103; GenAI nonusers: n=1696.

c Overall: n= 2790; GenAI users: n=1101; GenAI nonusers: n=1689.

d Overall: n= 2789; GenAI users: n=1099; GenAI nonusers: n=1690.

e Overall: n= 2782; GenAI users: n=1099; GenAI nonusers: n=1683.

f Overall: n= 2777; GenAI users: n=1102; GenAI nonusers: n=1675.

g ID: infectious disease.

h Overall: n= 2812; GenAI users: n=1106; GenAI nonusers: n=1706.

i Overall: n= 2799; GenAI users: n=1102; GenAI nonusers: n=1697.

j Overall: n= 2795; GenAI users: n=1103; GenAI nonusers: n=1692.

k Overall: n= 2772; GenAI users: n=1092; GenAI nonusers: n=1680.

l Overall: n= 2776; GenAI users: n=1095; GenAI nonusers: n=1681.

m Overall: n= 2770; GenAI users: n=1092; GenAI nonusers: n=1678.

n Overall: n= 2765; GenAI users: n=1091; GenAI nonusers: n=1674.

o Overall: n= 2768; GenAI users: n=1092; GenAI nonusers: n=1676.

p Overall: n= 2774; GenAI users: n=1095; GenAI nonusers: n=1679.

Similarly, users more often recognized essential competencies for ID management: history taking and physical examination (965/1106, 87.3% vs 1296/1706, 76%; *P*<.001), pathophysiological knowledge (958/1102, 86.9% vs 1286/1697, 75.8%; *P*<.001), and clinical reasoning (951/1103, 86.2% vs 1301/1692, 76.9%; *P*<.001).

### Affective Domain of GenAI Use Among Resident Physicians

Overall, 57.3% (1584/2765) to 60.1% (1663/2768) of respondents assessed GenAI for effectiveness, safety, applicability, and validity, while 47.3% (1311/2770) and 49.8% (1381/2774) considered fairness and transparency, respectively. Across all domains, users showed greater ethical awareness than nonusers: effectiveness (764/1092, 70% vs 843/1680, 50.2%; *P*<.001), safety (755/1095, 68.9% vs 892/1681, 53.1%; *P*<.001), fairness (615/1092, 56.3% vs 696/1678, 41.5%, *P*<.001), applicability (740/1091, 67.8% vs 844/1674, 50.4%; *P*<.001), validity (772/1092, 70.7% vs 891/1676, 53.1%; *P*<.001), and transparency (645/1095, 58.9% vs 736/1679, 43.8%; *P*<.001).

### Association Between GenAI Use and the Management of ID

GenAI use was independently associated with favorable behaviors (Table 3): thorough patient examination (adjusted odds ratio [aOR] 1.42, 95% CI 1.18‐1.70), appropriate antimicrobial selection (aOR 1.57, 95% CI 1.32‐1.87), familiarity with current epidemic information (aOR 1.46, 95% CI 1.22‐1.75), local antibiogram use (aOR 1.62, 95% CI 1.38‐1.89), individualized goal setting (aOR 1.64, 95% CI 1.40‐1.92), and individualized treatment strategy (aOR 1.45, 95% CI 1.23‐1.71).

**Table 3.** Association between the use of generative AI (GenAI) as a search engine and resident physicians’ and bedside behaviors regarding the management of infectious diseases (n=2850^a^).

Variables	GenAI users, n (%)	GenAI nonusers, n (%)	Adjusted odds ratio (95% CI)^b^	*P* value
I examine patients thoroughly when suspecting infectious diseases^c^	861 (78.7)	1221 (72.2)	1.42 (1.18-1.70)	<.001
I choose antimicrobials based on patient characteristics, suspected foci, and suspected organisms^d^	821 (75.7)	1122 (66.5)	1.57 (1.32-1.87)	<.001
I am familiar with information on the current epidemics^e^	286 (26.3)	334 (19.8)	1.46 (1.22-1.75)	<.001
I refer to the local antibiogram to choose antimicrobials^f^	563 (51.5)	668 (39.6)	1.62 (1.38-1.89)	<.001
I set patient goals explicitly when the cure is deemed unattainable^g^	550 (50.4)	645 (38.4)	1.64 (1.40-1.92)	<.001
I consider the patient social background to determine the treatment strategy^h^	743 (68.2)	1009 (60.1)	1.45 (1.23-1.71)	<.001

a Responses from some study participants are missing.

b Adjusted odds ratios for GenAI use against GenAI nonuse were presented. Each estimate is an adjusted odds ratio in a multiple logistic regression model involving sex, postgraduate year, university or community hospital, infectious disease department rotation, the presence of antimicrobial stewardship programs, and overall General Medicine In-Training Examination score as covariates.

c Overall: n= 2785; GenAI users: n=1094; GenAI nonusers: n=1691.

d Overall: n= 2773; GenAI users: n=1085; GenAI nonusers: n=1688.

e Overall: n= 2772; GenAI users: n=1088; GenAI nonusers: n=1684.

f Overall: n= 2782; GenAI users: n=1094; GenAI nonusers: n=1688.

g Overall: n= 2772; GenAI users: n=1092; GenAI nonusers: n=1680.

h Overall: n= 2768; GenAI users: n=1089; GenAI nonusers: n=1679.

### Sensitivity Analysis

A sensitivity analysis using an alternative cutoff for dichotomization of item responses was performed, which largely demonstrated consistency with the primary analysis regarding the trends of demographics, the 3 domains of GenAI, and the association between GenAI use and bedside behaviors. Statistically significant differences diminished in only 3 items: concerns over potential knowledge collapse and inadequate skill development, awareness of the necessity for transparency, and acknowledgment of the importance of clinical reasoning ability (MultimediaAppendices 1, 3  4).

## Discussion

### Principal Findings

In this nationwide questionnaire survey of resident physicians in Japan conducted in January 2025, 39.4% (1124/2850) of respondents reported using GenAI as a search engine, although textbooks remained their primary educational resource. Perceived GenAI literacy and ethical considerations varied, with nonusers demonstrating lower familiarity. Fewer than half of respondents recognized the importance of disclosing GenAI use to patients. Notably, GenAI use was positively associated with preferred behaviors in the management of IDs.

### Comparison to Prior Work

Despite its potential, the penetration of GenAI use as a search engine remained limited, both quantitatively and qualitatively, among resident physicians in Japan as of 2025. Consistent with earlier studies of physicians in training in the United States (2023) [24], Türkiye (2023‐2024) [25], and China (2024) [26], fewer than half of the respondents used GenAI in clinical practice. Furthermore, even among GenAI users, GenAI’s role as a search engine was peripheral, as most of them primarily used other tools. These findings may be at least partially explained by the unavailability of training for the appropriate application of GenAI [27]. Although GenAI offers opportunities for self-study, its output may be unsuitable for learners with differing levels of knowledge to interpret reliably [28]. Accordingly, structured education appears necessary to prepare resident physicians for the integration of GenAI into clinical practice.

Our study showed the systematic differences between GenAI users and nonusers. First, we found a lower likelihood of GenAI use among female resident physicians compared with their male counterparts. A study of physicians in training in the United States suggested a trend of less interest in GenAI among female than male respondents [24]. Probably, in the process of adopting new technology, female physicians prioritize social influence from their peers over its performance expectancy to determine whether to accept it [27]; they may defer their acceptance until GenAI is used by their peers. Second, nonusers consistently exhibited limited perceived GenAI literacy. According to the diffusion of innovations theory, the “early and late majority”—those likely to adopt GenAI in the future—tend to rely more on peer behavior (eg, colleagues’ GenAI use) than on theoretical understanding (eg, GenAI mechanisms), in contrast to “early adopters” (ie, current users) [29].

Our findings highlighted that transparency with patients was among the least considered aspects of GenAI use, although many respondents recognized the limitations of GenAI, such as confabulations and the possibility of biased outputs. The tendency of physicians to avoid disclosing GenAI use to patients has also been observed outside Japan [24] and may conflict with the principle that high-quality care should remain patient-centered [30]. From the patient’s perspective, ensuring transparency and explainability in the clinical decision-making process is critical [23], particularly as patients have begun voicing concerns about potential physician overreliance on GenAI [31,32].

In the present study, our hypothesis of knowledge collapse and inadequate skill development associated with GenAI use among resident physicians was not supported. Although this finding may be reassuring, the association may have been affected by several factors. First, unmeasured GenAI users’ traits, such as inquisitiveness, may have influenced the results. Second, other anticipated roles of GenAI, such as democratizing and accelerating access to context-specific medical knowledge, may have obscured the associations [33,34]. Nevertheless, empirical evidence regarding the long-term impact of GenAI on physicians’ performance remains limited, underscoring the need for further investigation.

### Limitations

This study has several limitations. First, the cross-sectional design precludes assessment of causal relationships between GenAI use and physicians’ knowledge, attitudes, or behaviors. The associations among these factors may also have been affected by unmeasured variables, such as learners’ inquisitiveness and diligence, although we tried to mitigate their impact by including the GM-ITE score in our model. Longitudinal studies at multiple time points are required to capture changes and inform the timely development of medical education. Second, nonresponse bias may have influenced the findings, potentially leading to the overrepresentation of GenAI-familiar resident physicians. Although we suggested that GenAI literacy was insufficient among resident physicians, we may have underestimated the severity of the issue. Third, the validity and reliability of our original survey items have yet to be explored, although we conducted a pilot test to enhance face validity. Specifically, we measured perceived GenAI literacy via a survey as a surrogate for actual GenAI literacy, which requires direct observation in clinical settings. Given the possibility that some respondents who misunderstood GenAI literacy reported their awareness, our study findings may have overestimated the level of GenAI literacy among resident physicians. Finally, we analyzed most 5-point Likert scale items after dichotomizing them to improve interpretability. Therefore, we may have overlooked nuanced differences.

### Future Directions

Our study findings underscore the need for residency programs to provide structured education on GenAI use in clinical contexts [6]. Such training should address not only effective prompt construction [35] but also the cultivation of critical appraisal skills to avoid premature closure [7,36] and the promotion of ethical considerations, including transparency with patients during patient care, all of which may need to be incorporated into future residency competency frameworks. The approach aligns with the recently proposed DEFT (diagnosis, evidence, feedback, and teaching)–AI framework for improving the quality of bedside GenAI education [7].

### Conclusions

Resident physicians in Japan currently use GenAI as a search engine to a limited extent. Their literacy regarding GenAI remains inadequate, particularly among nonusers.

## Supplementary material

10.2196/89750 Multimedia Appendix 1 Association between the use of generative AI as a search engine and resident physicians’ demographics and educational resources using a different cutoff.

10.2196/89750 Multimedia Appendix 2 Resident physicians’ perceived generative AI literacy in the cognitive and affective domains.

10.2196/89750 Multimedia Appendix 3 Association between the use of generative AI (GenAI) as a search engine and resident physicians’ perceived GenAI literacy in cognitive and affective domains using a different cutoff (n=2850).

10.2196/89750 Multimedia Appendix 4 Association between the use of generative AI as a search engine and resident physicians’ bedside behaviors regarding the management of infectious diseases using a different cutoff.

## Abbreviations

aOR: adjusted odds ratio
DEFT: diagnosis, evidence, feedback, and teaching
FAVES: fairness, appropriateness, validity, effectiveness, and safety
GenAI: generative AI
GM-ITE: General Medicine In-Training Examination
ID: infectious disease
JAMEP: Japan Institute for Advancement of Medical Education Program
PGY: postgraduate year
STROBE: Strengthening the Reporting of Observational Studies in Epidemiology
