# Supplementary material for: Current Landscape of Generative AI Use as a Search Engine Among Resident Physicians: Cross-Sectional Study
Source: JMIR AI. 2026 Jul 17;5:e89750. doi: 10.2196/89750 (PMC13378904; doi:10.2196/89750)
Supplement: Multimedia Appendix 1 [file ai-v5-e89750-s001.pdf]

Supplementary Table 1. The association between the use of generative artificial intelligence as a search engine and resident physicians' demographics and educational resources, using a different cut-off

Variables	GenAI users (n = 1,899)	Non-GenAI users (n = 951)	*P* value
**Participants demographics**			
Sex (n = 2,850)			0.01
Male	1,287 (67.8)	600 (63.1)	
Female	612 (32.2)	351 (36.9)	
PGY (n = 2,850)			0.47
PGY-1	958 (50.4)	466 (49.0)	
PGY-2	941 (49.6)	485 (51.0)	
Age under 30 (n = 2,714[a])	1,624 (89.7)	811 (89.2)	0.54
Institution type (n = 2,850)			0.54
University hospital	359 (18.9)	189 (19.9)	
Community hospital	1,540 (81.1)	762 (80.1)	
**Educational resources**			
I have received training on the application of GenAI as a search engine (n = 2,837[a]).	838 (44.4)	215 (22.7)	<.001
I rely on self-study instead of seeking advice from an attending physician to address questions (n = 2,824[a]).	1,222 (65.2)	518 (54.6)	<.001
What resources do you primarily use to address questions concerning **unfamiliar diseases**? (n = 2,827[a])			
Printed or e-textbooks	740 (39.3)	410 (43.4)	

Medical apps	531 (28.2)	231 (24.4)
Internet search without employing GenAI	386 (20.5)	250 (26.5)
GenAI-generated articles or summaries	154 (8.2)	14 (1.5)
Review articles	46 (2.4)	28 (3.0)
Original articles	25 (1.3)	12 (1.3)
What resources do you primarily use to address questions concerning **familiar diseases**? (n = 2,819[a])		
Printed or e-textbooks	1,002 (53.4)	565 (59.9)
Medical apps	456 (24.3)	196 (20.8)
Internet search without employing GenAI	245 (13.1)	154 (16.3)
GenAI-generated articles or summaries	123 (6.6)	11 (1.2)
Review articles	35 (1.9)	14 (1.5)
Original articles	14 (0.7)	4 (0.5)
What resources do you primarily use to address **differential diagnoses**? (n = 2,817[a])		
Printed or e-textbooks	1,029 (54.9)	582 (61.7)
Medical apps	387 (20.7)	192 (20.3)
Internet search without employing GenAI	245 (13.1)	148 (15.7)
GenAI-generated articles or summaries	175 (9.3)	13 (1.4)
Review articles	27 (1.4)	5 (0.5)
Original articles	10 (0.5)	4 (0.4)

Note:

Data are presented as a number (%) unless otherwise specified.

Abbreviations: GenAI, generative artificial intelligence; PGY, postgraduate year.

[a]Responses from some study participants are missing.
