# Supplementary material for: Current Landscape of Generative AI Use as a Search Engine Among Resident Physicians: Cross-Sectional Study
Source: JMIR AI. 2026 Jul 17;5:e89750. doi: 10.2196/89750 (PMC13378904; doi:10.2196/89750)

Supplementary Figure 1. Resident physicians' perceived generative artificial intelligence literacy in cognitive and affective domains.

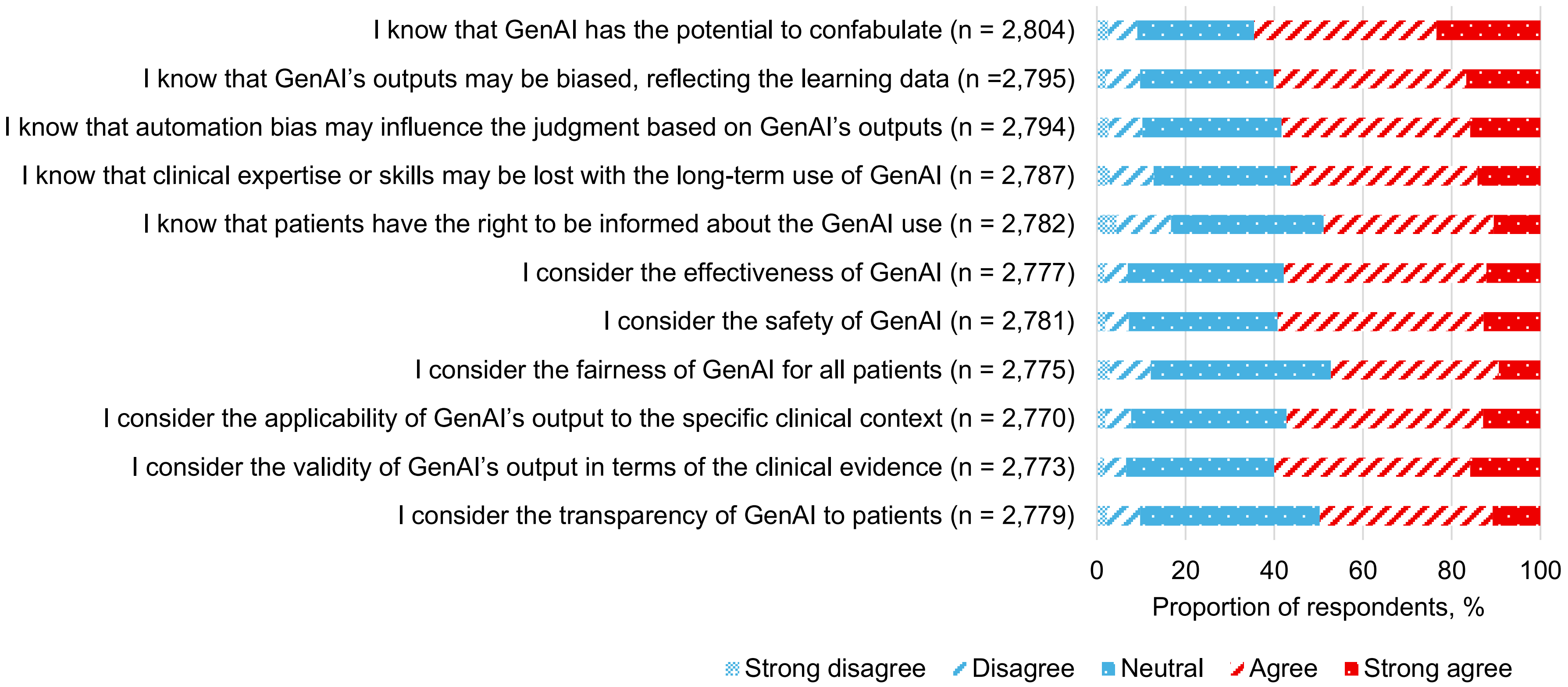

Supplement: Multimedia Appendix 2 [file ai-v5-e89750-s002.pdf]
