# Supplementary material for: Current Landscape of Generative AI Use as a Search Engine Among Resident Physicians: Cross-Sectional Study
Source: JMIR AI. 2026 Jul 17;5:e89750. doi: 10.2196/89750 (PMC13378904; doi:10.2196/89750)
Supplement: Multimedia Appendix 3 [file ai-v5-e89750-s003.pdf]

Supplementary Table 2. The association between the use of generative artificial intelligence as a search engine and resident physicians' perceived generative artificial intelligence literacy in cognitive and affective domains, using a different cut-off (n = 2,850)

Variables	GenAI users (n = 1,899)	Non-GenAI users (n = 951)	*P* value
**Cognitive domain**			
I know that GenAI has the potential to confabulate (n = 2,799[a])	1,725 (92.6)	818 (87.3)	<.001
I know that GenAI's outputs may be biased, reflecting the learning data (n =2,790[a])	1,710 (92.2)	809 (86.5)	<.001
I know that automation bias may influence the judgment based on GenAI's outputs (n = 2,789[a])	1,702 (91.7)	803 (86.1)	<.001
I know that clinical expertise or skills may be lost with the long-term use of GenAI (n = 2,782[a])	1,618 (87.3)	808 (87.0)	0.80
I know that patients have the right to be informed about the GenAI use (n = 2,777[a])	1,554 (84.0)	760 (82.1)	0.21
I think that history-taking and physical examination remains crucial in the management of ID (n = 2,812[a]).	1,815 (97.2)	895 (94.8)	.002
I think that understanding pathophysiology remain crucial in the management of ID (n = 2,799[a]).	1,818 (97.7)	898 (95.7)	.004
I think that clinical reasoning ability remains crucial in the management of ID (n = 2,795[a]).	1,810 (97.3)	911 (97.4)	0.85
**Affective domain**			
I consider the effectiveness of GenAI (n = 2,772[a])	1,756 (95.2)	825 (89.0)	<.001
I consider the safety of GenAI (n = 2,776[a])	1,737 (94.1)	840 (90.2)	<.001
I consider the fairness of GenAI for all patients (n = 2,770[a])	1,640 (89.0)	793 (85.5)	.007

I consider the applicability of GenAI's output to the specific clinical context (n = 2,765[a])	1,717 (93.4)	836 (90.3)	.004
I consider the validity of GenAI's output in terms of the clinical evidence (n = 2,768[a])	1,747 (94.7)	840 (90.0)	<.001
I consider the transparency of GenAI to patients (n = 2,774[a])	1,695 (91.8)	809 (87.3)	<.001

Note:

Data are presented as a number (%) unless otherwise specified.

Abbreviations: GenAI, generative artificial intelligence; ID, infectious diseases; PGY, postgraduate year.

[a]Responses from some study participants are missing.
