# Supplementary material for: Current Landscape of Generative AI Use as a Search Engine Among Resident Physicians: Cross-Sectional Study
Source: JMIR AI. 2026 Jul 17;5:e89750. doi: 10.2196/89750 (PMC13378904; doi:10.2196/89750)
Supplement: Multimedia Appendix 4 [file ai-v5-e89750-s004.pdf]

Supplementary Table 3. The association between the use of generative artificial intelligence as a search engine and resident physicians' and bedside behaviors regarding the management of infectious diseases, using a different cut-off

Variables	Adjusted odds ratio for GenAI use[a]	*P* value
**Behaviors associated with the management of infectious diseases**		
I examine patients thoroughly when suspecting infectious diseases (n = 2,785[b]).	1.53 (1.12–2.11)[c]	.008
I choose antimicrobials based on patient characteristics, suspected foci, and suspected organisms (n = 2,773[b]).	1.49 (1.11–2.02)[c]	.009
I am familiar with information on the current epidemics (n = 2,772[b]).	1.64 (1.40–1.93)[c]	<.001
I refer to the local antibiogram to choose antimicrobials (n = 2,782[b]).	1.61 (1.35–1.92)[c]	<.001
I set patient goals explicitly when the cure is deemed unattainable (n = 2,772[b]).	1.50 (1.23–1.83)[c]	<.001
I consider the patient social background to determine the treatment strategy (n = 2,768[b]).	1.62 (1.24–2.11)[c]	<.001

Note:

Abbreviations: GenAI, generative artificial intelligence.

[a]Adjusted odds ratios for GenAI use against non-GenAI use were presented.

[b]Responses from some study participants are missing.

[c]Each estimate is an adjusted odds ratio in a multiple logistic regression model involving sex, postgraduate year, university or community

hospital, infectious disease department rotation, the presence of antimicrobial stewardship programs, and overall General Medicine In-Training Examination score as covariates.
